# Insights into the Structure, Function, and Ligand Discovery of the Large Neutral Amino Acid Transporter 1, LAT1

**DOI:** 10.3390/ijms19051278

**Published:** 2018-04-24

**Authors:** Natesh Singh, Gerhard F. Ecker

**Affiliations:** Department of Pharmaceutical Chemistry, University of Vienna, Althanstrasse 14, 1090 Wien, Austria; singh.natesh@gmail.com

**Keywords:** amino acid transporter, LAT1, AdiC, anticancer, melphalan, covalent inhibitor

## Abstract

The large neutral amino acid transporter 1 (LAT1, or SLC7A5) is a sodium- and pH-independent transporter, which supplies essential amino acids (e.g., leucine, phenylalanine) to cells. It plays an important role at the Blood–Brain Barrier (BBB) where it facilitates the transport of thyroid hormones, pharmaceuticals (e.g., l-DOPA, gabapentin), and metabolites into the brain. Moreover, its expression is highly upregulated in various types of human cancer that are characterized by an intense demand for amino acids for growth and proliferation. Therefore, LAT1 is believed to be an important drug target for cancer treatment. With the crystallization of the arginine/agmatine antiporter (AdiC) from *Escherichia Coli*, numerous homology models of LAT1 have been built to elucidate the substrate binding site, ligand–transporter interaction, and structure–function relationship. The use of these models in combination with molecular docking and experimental testing has identified novel chemotypes of ligands of LAT1. Here, we highlight the structure, function, transport mechanism, and homology modeling of LAT1. Additionally, results from structure–function studies performed on LAT1 are addressed, which have enhanced our knowledge of the mechanism of substrate binding and translocation. This is followed by a discussion on ligand- and structure-based approaches, with an emphasis on elucidating the molecular basis of LAT1 inhibition. Finally, we provide an exhaustive summary of different LAT1 inhibitors that have been identified so far, including the recently discovered irreversible covalent inhibitors.

## 1. Introduction 

LAT1 (SLC7A5) is a sodium and pH-independent transmembrane transporter that forms a heterodimeric complex with the glycoprotein 4F2hc (CD98, SLC3A2) to import large and neutral amino acids (e.g., leucine, phenylalanine) in exchange for intracellular amino acids (e.g., glutamine) [1,2]. It is highly expressed in cells that require a continuous supply of amino acids, such as neural, glial, placental cells, activated T cells, and endothelial cells of the Blood–Brain Barrier (BBB) [3,4,5]. At the BBB, LAT1 is stereospecific (l > d) [6] and exhibits higher affinity for amino acids compared to LATs in the peripheral tissues [7]. It preferentially transports branched amino acids in the following order: Phe > Trp > Leu > Ile > Met > His > Tyr > Val [3]. It has been shown that LAT1 has higher affinity for intracellular amino acids as compared to the extracellular ones, indicating that the transport rate is controlled by the concentration of intracellular substrates [8]. Recently, it has been shown that LAT1 is the sole transport-competent unit, while 4F2hc does not play any significant role in the intrinsic transport function [9]. The glycoprotein 4F2hc functions as a molecular chaperone enabling LAT1 [10] to reach its definitive localization in the cellular membrane. In the BBB, LAT1 facilitates the transport of nutrients and drugs into the central nervous system (CNS). Thus, it is an interesting target for the design and optimization of compounds that show poor or sub-optimal BBB permeability, as it might serve as an uptake transporter. It has also been demonstrated that a homozygous global knockout of LAT1 in mice is embryonically lethal, which may perhaps be due to its critical role at the placental barrier where it allows the uptake of essential amino acids required for the growth of the fetus [11].

Furthermore, LAT1 expression is highly upregulated in many tumor cell lines [12,13,14] and human cancers, such as breast, prostate, lung, colorectal, head, neck, and gliomas [15,16,17,18,19,20], where it has been shown to play a substantial role in growth and survival. The functional significance of LAT1 in the growth of tumors has been demonstrated through genetic manipulation, whereby knockdown of LAT1 with RNA interference (RNAi) [21,22,23,24,25] and its genetic disruption by zinc fingers nucleases-mediated gene knockout [26] in cancer cells exhibited reduced leucine uptake and cell proliferation. Therefore, LAT1 has been considered as a potential drug target by which the growth and proliferation of cancer cells could be reduced [27]. LAT1 inhibitors act by depriving the cancer cells of amino acids, thus prohibiting protein synthesis and cell proliferation. However, the amino acid leucine has been shown to stimulate protein synthesis and accelerate cell growth by regulating mammalian target of rapamycin complex 1 (mTORC1), and LAT1 inhibition is described to suppress mTORC1 signaling and consequently cancer growth [28,29]. The compound 2-Amino-2-norbornanecarboxylic acid (BCH) is regarded as a classical LAT inhibitor which reduces cancer cell growth and induces apoptosis [30,31,32].

With the crystallization of arginine/agmatine antiporter (AdiC), numerous homology models of LAT1 have been reported and are proving invaluable in the study of the transporter structure and mechanism of amino acid transport. These models have guided the elucidation of the substrate binding site, inter- and intra-molecular interactions, substrate translocation mechanism, and conformational states of the protein. The virtual screening efforts have enabled the identification of novel ligands, indicating that AdiC-based LAT1 models can be useful for structure–function studies. This article highlights the structure, function, transport mechanism, and in silico studies of LAT1.

## 2. Structure of LAT1–4F2hc

**Figure 1:**
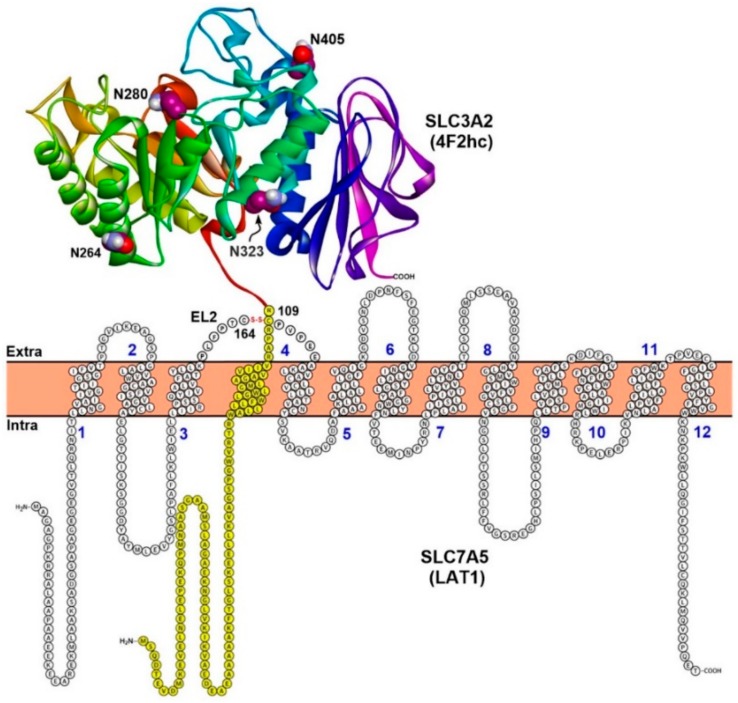
Topology model of LAT1–4F2hc. LAT1 consist of 12 putative transmembrane segments (TMs) and it is associated with 4F2hc through a conserved disulfide bridge between C164 and C109. The protein 4F2hc is a type II membrane *N*-glycoprotein (one TM, in yellow; four glycosylation sites in the extracellular domain: N264, N280, N323, and N405 are shown in space-filling style with carbon atoms colored maroon) with an intracellular N-terminus and an extracellular C-terminus. In contrast, LAT1 has intracellular N-terminus and C-terminus and is not glycosylated. The crystal structure of the extracellular domain (ED) of human 4F2hc (PDB ID: 2DH2) [38] is shown in ribbon representation. This figure was recreated from reference [2].

LAT1 is a member of Heterodimeric Amino acid Transporters (HATs) that are composed of a light chain (SLC7) that mediates the transport of amino acids, and a heavy chain (SLC3) that catalyzes plasma membrane localization and stabilization of the light chains. LAT1 consist of 12 putative transmembrane segments (TMs) arranged in two layers. The inner layer consists of TM1, TM3, TM6, TM8, and TM10, and is surrounded by the outer layer, comprising TM2, TM4, TM5, TM7, TM9, TM11, and TM12. The N- and C-terminal ends of LAT1 are localized intracellularly, while 4F2hc has the N- and C-terminal localized intracellularly and extracellularly, respectively [1,33] (Figure 1). The protein 4F2hc is an *N*-glycosylated protein (four putative *N*-glycosylation sites: N264, 280, 323, 405) with one TM domain and one large extracellular domain (ED). LAT1 and 4F2hc are covalently linked by a disulfide bond between the cysteines located a few residues away from the TM of the heavy subunit and in the putative extracellular loop 2 (EL2) of the light subunit between TM3 and TM4 (Figure 1). LAT1 is predicted to be covered extracellularly by the extracellular domain of 4F2hc, and both proteins are involved in putative non-covalent interactions. Rosell et al. [34] showed via protein–protein docking that the desolvation energy of hydrophobic residues contributes mainly to the binding of 4F2hc-ED and LAT2, suggesting that LAT1 and 4F2hc-ED might be involved in similar interactions. In a further study, mutation of the cysteines responsible for the disulfide bond did not break the interaction in y^+^L–4F2hc [35] indicating favorable interactions between the heavy and the light subunit. The closest prokaryotic homolog of LAT1 is the serine/threonine antiporter (SteT), with which it shares a sequence identity of ~30% [36,37].

## 3. Structure of LAT1 Homolog AdiC

**Figure 2:**
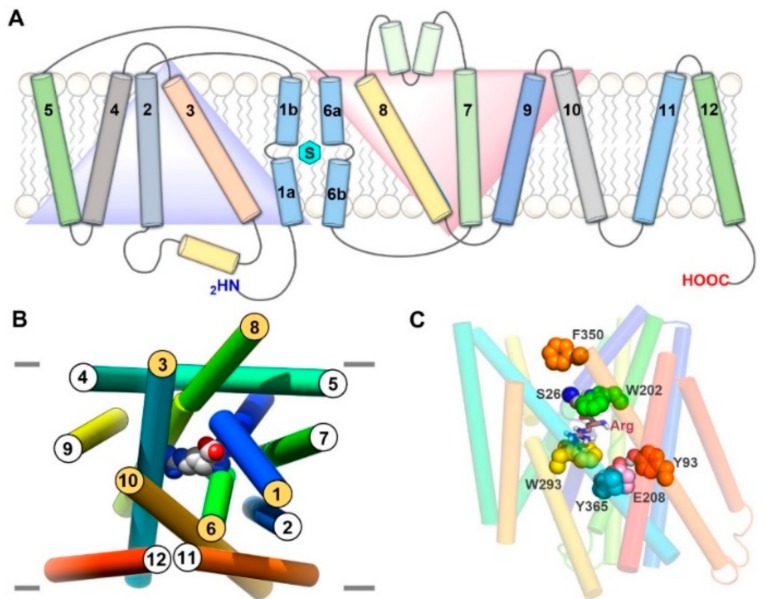
(**A**) A 2D representation of the AdiC in lipid bilayer. TMs (shown as cylinders) 1–5 and 6–10 are symmetrical but with inverted motifs (blue and red triangles); the bound substrate is depicted as a cyan hexagon; (**B**) Perpendicular periplasmic view of arginine-bound AdiC showing the 3D arrangement and orientation of different TMs. The arginine bound to AdiC is shown in space-filling representation; (**C**) Front view of arginine-bound AdiC showing functionally essential gate residues in AdiC. Arginine (Arg) is displayed in stick style, and gate residues are shown in space-filling form. The carbon atoms of the gate residues are colored blue (S26), green (W202), yellow (W293), cyan (Y365), pink (E208) and orange (Y93, F350).

**Figure 3:**
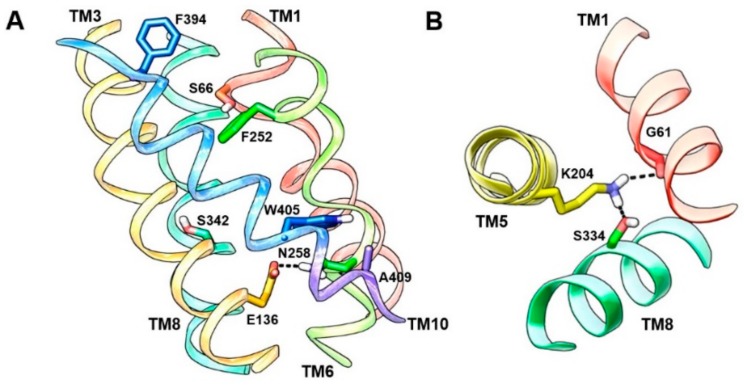
(**A**) Putative gate residues are depicted in the outward-occluded model of LAT1. F394 is the front doorway residue that corresponds to F350 of AdiC. S66 and F252 (proximal gate residues) of LAT1 correspond to S26 and W202 of AdiC. S342 (middle gate residue) of LAT1 corresponds to W293 of AdiC. N258, E136, and A409 (distal gate residues) of LAT1 correspond to E208, Y93, and Y365 of AdiC. The hydrogen bond interaction between E136 and N258 indicates a closed distal gate; (**B**) Hydrogen bond interactions of K204 (TM5) with G61 (TM1) and S334 (TM8) in the inward-open model of LAT1 based on the ApcT template (PDB ID: 3GI9) [40].

**Figure 4:**
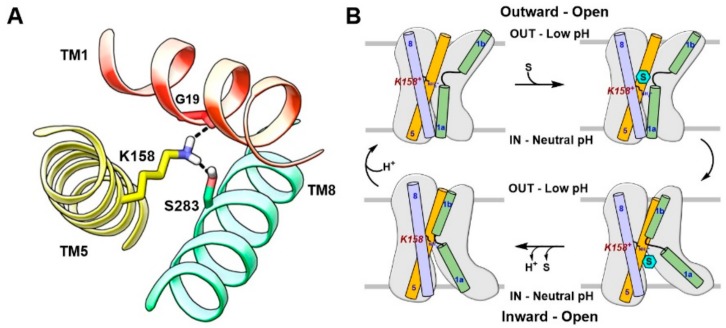
(**A**) Hydrogen bond interactions of K158 (TM5) with G19 (TM1) and S283 (TM8) in ApcT (PDB ID: 3GI9) [40]; (**B**) Proposed mechanism of the conformational transition in ApcT. ApcT adopts an inward-open, occluded conformation when K158 is neutral (bottom left). An acidic pH stimulates the protonation of K158 leading to an outward-open state (top left). Upon substrate binding (top right), the transporter isomerizes to an inward-open state (bottom right). The formation of the inward-open, occluded conformation is preceded by the release of the substrate and proton(s) into the cytoplasm; (**B**) The figure was recreated from reference [40].

The arginine-bound AdiC crystal structure (PDB ID: 3L1L) [39] in an outward-occluded conformation is a reliable template for molecular modeling of LAT1. AdiC is a distant but credible LAT1 homolog, with ~20% sequence identity and ~40% sequence similarity. Apart from AdiC, LAT1 shows a sequence identity of ~22% and a sequence similarity of ~41% to the broad-specificity amino acid transporter (ApcT) from *Methanocaldococcus jannaschii* and a sequence identity of ~19% and a sequence similarity of ~35% to the glutamate/γ-aminobutyric acid (GABA) antiporter (GadC) from *Escherichia coli*, whose X-ray structures are also available [40,41]. Despite the low sequence identity, LAT1 shares the same fold of AdiC and leucine transporter (LeuT). The AdiC structure revealed an inverted symmetry similar to LeuT between TM1–5 and TM6–10, while TM11 and TM12 are located far from the substrate binding site (Figure 2A). The TM1 and TM6 α-helices are unwound at their approximate midpoints, near the center of the lipid bilayer, to harbor the substrate binding site. The binding site is surrounded by five TMs: 1, 3, 6, 8, and 10 (Figure 2B), and access to this site is modulated by residues that are located within the transport path, with some residues directly contributing to substrate recognition. These amino acids can be grouped into three layers. The layer proximal to the periplasm (the proximal layer) consists of S26 in TM1 and W202 in TM6. The middle layer is represented by W293 in TM8. The segment distal to the periplasm (the distal layer) has three amino acids, i.e., Y93 in TM3, E208 in TM6, and Y365 in TM10 (Figure 2C) [42]. It has been proposed that these three layers function as three gates that control the transport of substrate amino acids [42]. W202 likely serves as the proximal gate element that remains open in the substrate-free state and closes upon binding of extracellular substrates. The substrates are bound between W202 and the middle gate W293. The opening of the central gate involves the translocation of W293 and a conformational shift of TM8, allowing the substrate to ‘sink’ and contact the distal gate. Such contact is proposed to disrupt the hydrogen bond interactions between E208 and Y93/Y365, inducing further changes in TM3 and TM10 and leading to an inward-open conformation for the exchange of substrate. These putative gating residues are highly conserved among AdiC, cadaverine/lysine antiporter (CadB), and ornithine/putrescine antiporter (PotE), indicating a conserved transport mechanism [42]. Through an insightful investigation of the alternating access mechanism in AdiC using classical and targeted molecular dynamics, Krammer et al. [43] established the functional importance of critical residues involved in the binding and translocation of arginine. The study demonstrated that arginine is first grabbed by F350 and subsequently by the proximal gate element W202. The side chain rotation of W202 stimulates the outward-occluded conformation, and arginine gets sandwiched between W202 and the middle gate W293. This closure is characterized by a strong π–π interaction between F350 and W202, indicating that F350 is probably serving as an upper front door residue preceding the proximal gate W202 (Figure 2C). The unbinding of arginine from the outward-occluded state is driven by cation–π interactions of arginine with W293 and distal gate Y93 and by an ionic interaction with distal gate E208. This set of interactions breaks the hydrogen bond interaction between Y93 and E208, triggering the release of arginine into the cytosol through a rotamer transition of W293 leading to an inward-open state. Moreover, molecular dynamics (MD) simulations demonstrated that synchronised reorganizations of the TM1 and TM6 backbone segments are essential for the conformational evolution from the outward-open to the inward-open state in AdiC [43]. The various gating residues of AdiC correspond to F394 (TM10) (front doorway residue), S66 (TM1) and F252 (TM6) (proximal gate), S342 (TM8) (middle gate), and E136 (TM3), N258 (TM6), and A409 (TM10) (distal gate) of LAT1 as per the published alignments [42,44] (Figure 3A). Interestingly, the predicted model of LAT1 shows a hydrogen bond interaction between E136 and N258 (Figure 3A), which is analogous to the hydrogen bond network among the distal residues of AdiC. The role of S342 and A409 as putative gating residues seems questionable because of the high sequence diversity of the corresponding TMs. Rather, the structural alignment of LAT1 and AdiC (PDB ID: 3L1L) revealed that the side chain of W405 of LAT1 is occupying a position equivalent to the side chain of Y365 of AdiC, indicating that W405 might be functioning as a distal residue (Figure 3A). It seems likely that LAT1 displays a transport mechanism similar to that of AdiC. Among all the gating residues, F252 has been experimentally confirmed as a proximal gate of LAT1 (see Section 6). Future experimental studies are required to assess the potential role of residues S66, E136, N258, S342, A409, and W405 in substrate binding and translocation of LAT1. Contrary to AdiC, the transport cycle in ApcT is supposed to be regulated by the protonation and deprotonation of K158 (TM5). The currently available structures of ApcT (PDB IDs: 3GIA, 3GI8, 3GI9) [40] represent the neutral and non-protonated state of K158, i.e., the inward-open apo yet occluded state of the transporter after unbinding of substrate/proton(s) into the cytoplasm. K158 is situated between TM1 and TM8 and is involved in hydrogen bond interactions with the backbone of G19 (TM1) and the side chain of S283 (TM8) (Figure 4A). Moreover, “there is a solvent-accessible path from the cytoplasmic solution to K158, between TM1 and TM5” [40]. It is suggested that upon protonation of K158, ApcT isomerizes from the inward-open state to the outward-open state (Figure 4B) through the formation of a kink in TM1 at a position analogous to the kink in LeuT, engaging the carbonyl oxygen of I22 (TM1) and the hydroxyl groups of S283 and S286 (TM8). Proton binding and unbinding in combination with the local conformational changes in TM1 trigger the movement of TM1a, TM1b, TM6a, and TM6b, thus facilitating the opening and closing of the extracellular and intracellular gates [40,45]. Interestingly, the hydrogen-bonded residues of ApcT are conserved in LAT1 and correspond to K204 (TM5), G61 (TM1), and S334 (TM8). Similar to K158 of ApcT, K204 is engaged in hydrogen bond interactions with G61 and S334 in LAT1 (Figure 3B). However, K204 seems buried and is not solvent-accessible in LAT1 compared to K158 in ApcT, as revealed by solvent-accessible surface area calculations. Therefore, K204 is possibly neutral and non-protonated, and most likely the conformational evolution is regulated by the interplay of gating residues in LAT1. The structural comparison of the inward-open structures, ApcT (PDB ID: 3GI9) and GadC (PDB ID: 4DJI) [41], revealed a pronounced conformational change within the transmembrane regions, i.e., TM1, TM6, TM7, and TM10. Since ApcT represents the inward-open yet occluded state, while GadC is in the inward-open state, this may provide an explanation for the conformational shift within the TMs. Additionally, the transport mechanism of GadC is believed to be different from that of ApcT. This might as well account for the conformational change in the two structures. Finally, we summarize that the potential templates available for comparative modeling of the principal states of the transport cycle of LAT1 consists of: (i) outward-open and substrate-free structures of AdiC (PDB IDs: 3LRB, 3LRC [42], 3NCY [46], 5J4I [47]); (ii) outward-open and substrate-bound structures of AdiC (PDB IDs: 5J4N [47], 3OB6 [48]); (iii) outward-occluded and substrate-bound structure of AdiC (PDB ID: 3L1L) [39]; (iv) inward-open, occluded structures of ApcT (PDB IDs: 3GIA, 3GI9, 3GI8) [40]; (v) inward-open structures of GadC (PDB IDs: 4DJK, 4DJI) [41].

## 4. Substrate Translocation Mechanism

**Figure 5:**
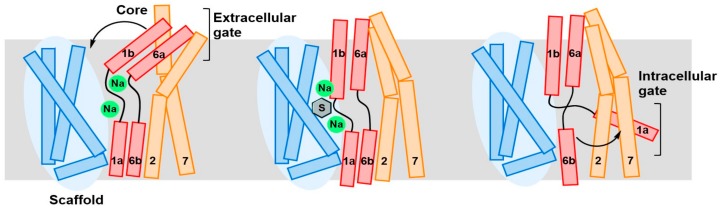
Schematic representation of the rocking-bundle alternating-access mechanism for the sodium-coupled amino acid symporter LeuT. The core domain is shown in red/orange, and the scaffold domain is in blue. Further local conformational changes of the extracellular TM1b, TM6a (red), and TM7 (orange), and intracellular TM1a define the outside and inside gates, respectively. The two sodium ions are shown as green spheres, and the substrate is shown as a grey hexagon. This figure was recreated from reference [56].

The most likely mechanism of substrate translocation across the cellular membrane for LAT1 and proteins having a “LeuT-like fold” is via the so-called “rocking-bundle alternating-access” mechanism. In this model, substrate binding between two architecturally discrete domains promotes the coupled movement of extracellular and intracellular gates around a centrally situated substrate binding site [49]. In the LeuT fold, TM3, TM4, TM8 and TM9 form the scaffold domain, while TM1, TM2, TM6 and TM7 represents the core domain [49,50]. The TM5 and TM10 serve as linkers between the two bundles. The opening of the outward-facing gate allows the binding of the substrate, which is then released into the cytoplasm by the opening of the intracellular barrier (Figure 5). Dickens et al. [51] derived a mathematical model to account for the alternate access mechanism in LAT1. In their investigation, kinetic data for LAT1-mediated transport of gabapentin in HEK293 cells served as input for a two-compartment model allowing an estimation for the Michaelis–Menten *V*_max_ and *K*_m_ parameters based on the alternative access mechanism. In another work, Napolitano et al. [52] proposed a random simultaneous transport mechanism in LAT1, which implies the formation of a ternary complex in which internal and external substrates are bound and translocated simultaneously towards opposite sides of the membrane. The basis for the simultaneous mechanism was attributed to the existence of an oligomeric structure of LAT1, where each monomer can bind and translocate substrates from the outside to the inside or vice versa, independently [53,54,55]. Because of the conservation of the LeuT fold, an alternative access mechanism seems highly likely in LAT1. However, an X-ray structure of LAT1 would be required to gain a better understanding of the transport mechanism.

## 5. Homology Modeling of LAT1

**Figure 6:**
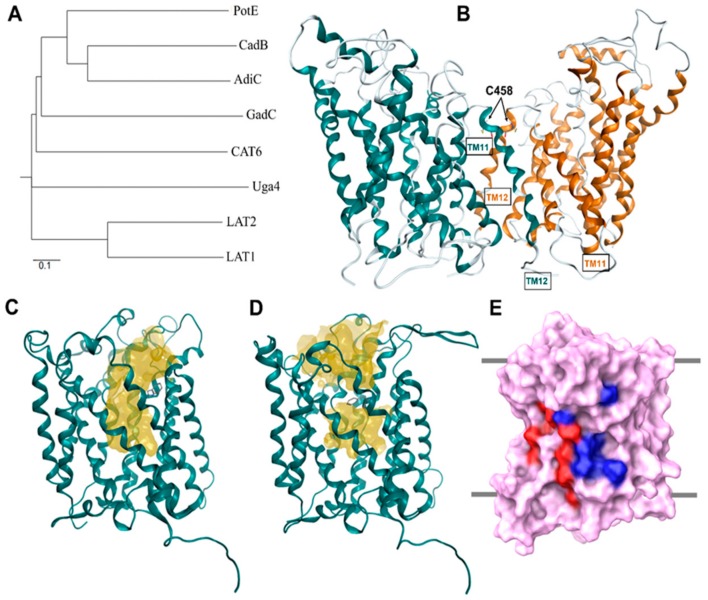
(**A**) Phylogenetic tree of amino acid transporters based on multiple sequence alignment [52]; (**B**) LAT1 homology model shown as ribbon representation of its dimeric form. Cysteine residues (C458) at the interface of the LAT1 dimer are shown in stick representation; (**C**) An outward-open model of LAT1; (**D**) An outward-occluded model of LAT1; the conserved residue F252 (stick representation) in LAT1 corresponding to W202 in AdiC behaves like a gate in LAT1, hindering the substrate access pathway (yellow surface), as shown in (**C**,**D**) [52]; (**E**) Front view of the LAT1 model showing the location of the putative cholesterol binding sites. Critical residues of the cholesterol/CHS binding sites I and II are rendered blue and red, respectively, on the molecular surface of LAT1 (purple). The multiple sequence alignment comprised amino acid sequences of LAT1 (UniProt accession number: Q01650, organism: Human), LAT2 (Q9UHI5, Human), Uga4 (P32837, *Saccharomyces cerevisiae*), Cat6 (Q9LZ20, *Arabidopsis thaliana*), GadC (P63235, *Escherichia coli*), AdiC (P60061, *E. coli*), CadB (P0AAE8, *E. coli*), and PotE (P0AAF1, *E. coli*). The multiple sequence alignment and phylogenetic tree data were generated using Clustal Omega [72]. The phylogenetic tree was created using the web server: http://iubio.bio.indiana.edu/treeapp/treeprint-form.html. The outward-occluded model of LAT1 was built using MODELLER v9.13 [57] and is based on the AdiC structure (PDB ID: 3L1L) and published alignment [44]. The amino acid sequence alignment was generated using PRIME [73] and was edited manually to match the published alignment. This alignment was then used to construct the homology model of LAT1. PyMol (version 1.6) [74] and Maestro [75] were used to visualize the structural models; (**B**–**D**) were adapted after securing permission from reference [52]. Copyright© 2018 Elsevier B.V.

With the crystallization of AdiC and ApcT, various LAT1 homology models have been built and studied. Details regarding the construction of homology models of LAT1 published to date are provided in Table 1. Geier et al. [44] constructed LAT1 in the outward-occluded conformation based on AdiC (PDB ID: 3L1L) and in an inward-apo state from the structure of the ApcT (PDB ID: 3GI9), by using MODELLER v9.11 [57]. Dickens et al. [51] modeled LAT1 based on AdiC (PDB ID: 3L1L) and ApcT (PDB ID: 3GIA), using the ITASSER server [58] that generates 3D atomic models from multiple threading alignments and iterative structural assembly simulations. PROMALS3D [59] was then used to obtain the sequence–structure alignment and for the identification of the putative LAT1 binding site. Napolitano et al. [52] constructed multiple sequence alignment involving three amino acid/polyamine/organocation (APC) antiporters, namely, AdiC, CadB, and PotE, one cationic amino acid transporter (CAT), CAT6, one amino acid/choline transporter (ACT), Uga4, one glutamate-GABA antiporter, GadC, the l-type amino acid transporter LAT2, and the target protein human LAT1. From the sequence alignment, it is evident that within the APC superfamily, AdiC shares considerable sequence homology with the GadC [60,61], CadB [62,63], and PotE [64,65,66] (Figure 6A). LAT1 models were constructed in an outward-open apo dimeric form against 3LRB (Figure 6B), an outward-open conformation in a holo monomeric form based on the template 3OB6 (Figure 6C), and an outward-occluded holo monomeric form against 3L1L (Figure 6D), using the PSIPRED Protein Sequence Analysis Workbench (http://bioinf.cs.ucl.ac.uk/psipred/) and the TMHMM Server (http://www.cbs.dtu.dk/services/TMHMM/).

It can be inferred from the published alignments [34,42,44,52] that evolutionarily conserved regions of LAT1 are primarily located in the TMs, while the extracellular and intracellular loops are less conserved. The N- and C-terminal regions are highly sequence-variable and are predicted to form long intracellular domains (Figure 1). The amino acid sequence alignment [42,44] shows short insertions of 1–3 residues within TM1, TM3, and TM4 of LAT1 indicating that the TMs of LAT1 are predicted to be longer than those of AdiC [44]. Additionally, long deletions are observed in TM9 and TM10 suggesting a high sequence diversity in these TMs, making the alignment in these regions unreliable [44]. As a result of these deletions, TM9 and TM10 have a discontinuous structure in the outward-occluded model of LAT1. However, these regions are far from the substrate binding site and are less likely to influence the active site conformation and the outcome of docking studies against this structure [44]. Contrary to the alignments presented in [42,44], the alignment provided in [34] does not show any gaps within TM1–12 because they were moved to the nearby loops through manual correction. However, this correction is accompanied by compromised sequence identity as compared to alignments [42,44,52] that might render the model uncertain, specifically for understanding the ligand–transporter interaction. Despite the low sequence identity, AdiC will continue to serve as a potential template for comparative modeling of LAT1 until the X-ray structures of new homologs become available.

**Figure 7:**
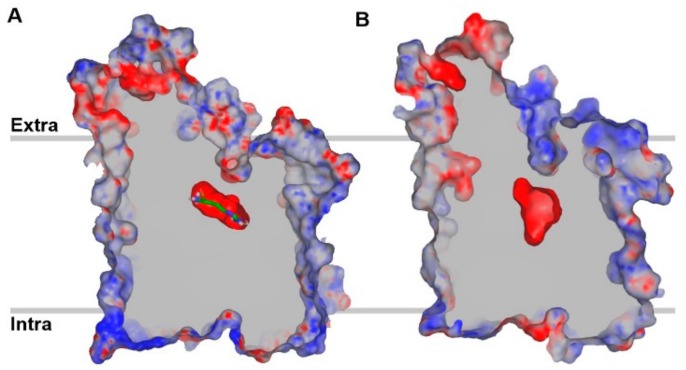
Centrally sliced structure of the electrostatic potential molecular surface of arginine-bound AdiC (**A**) and an outward-occluded model of LAT1 (**B**) showing the negative surface potential of the substrate binding cavity. The red and blue regions indicate negative and positive surface potential, respectively, while grey indicates the surface capping color. Arginine is shown in stick representation in (**A**). The binding pocket of LAT1 is predicted to be large and broad in size as compared to AdiC.

The TM1 and TM6 of LAT1 are predicted to be disrupted by short, nonhelical, glycine-containing loops. The GSG motif (TM1) G25–S26–G27 of AdiC corresponds to G65–S66–G67 in LAT1. The GVESA motif (TM6) G206–V–E–S–A210 of AdiC corresponds to G256–W–N–Y–L260 in LAT1. LAT1 is predicted to consist of a short re-entrant helix (9–10 residues long) between TM2 and TM3, sharing a YAY motif with AdiC. The substrate binding site of LAT1 is predicted to be located in an interior cavity of LAT1 within a substrate translocation pathway formed by the highly conserved regions of TM1, TM3, TM6, TM8, and TM10. The residues located in the helix break of TM1 and TM6 have exposed backbone groups that likely mediate the binding of substrates and assist in the conformational transition required for substrate translocation. Similar to AdiC, the binding site of the LAT1 is predicted to be negatively charged. However, LAT1 pocket is large and broad in size as compared to AdiC (Figure 7B). The comparison of the binding sites of the LAT1 and the AdiC structures provides an explanation for the differences in the substrate specificity of LAT1 and AdiC. LAT1 has several hydrophobic residues (I139, V148, F252, F402 and W405) located in the binding site, likely contributing to increased ligand binding affinity via favorable van der Waals contacts and hydrophobic interactions (π–π, alkyl). Some of these hydrophobic residues are replaced by non-hydrophobic residues in LAT1 homologs, including AdiC and other SLC7 members. For example, the aromatic W405 in LAT1 corresponds to the polar T361 in AdiC. Several large residues of AdiC are replaced by smaller residues in LAT1, creating a larger volume for binding, e.g., M104, I205, and W293 in AdiC correspond to the smaller V148, G255 and S342 in LAT1 [44].

Furthermore, experiments have provided evidence for the existence of an oligomeric structure of LAT1 and a possible involvement of C458 in a covalent interaction between the two monomers [37]. The homodimeric interface of the LAT1 model (Figure 6B) involves four TMs from each monomer. Hydrophobic amino acids in TM11 of one LAT1 molecule are predicted to be involved in favorable van der Waals interactions with hydrophobic residues in TM12 of the adjacent molecule. Because AdiC is a homodimer in detergent micelles and phospholipid membranes [67,68], the existence of a LAT1 homodimer is highly plausible.

Dickens et al. [69] have experimentally demonstrated that cholesterol modulates LAT1 stability and its transport activity. The depletion of cellular cholesterol resulted in reduced *V*_max_, but unaffected the *K*_m_ of LAT1-mediated uptake of l-DOPA in HEK293 cells, suggesting possible cholesterol interactions. LAT1 is predicted to have two cholesterol/cholesteryl hemisuccinate (CHS) binding sites (I/II), similar to the binding sites found in *Drosophila* dopamine transporter (dDAT) [70,71]. The alignment of dDAT and LAT1 sequences revealed that putative cholesterol-interacting residues in binding site I in LAT1 comprise residues L53 (TM1), V56 (TM1), V60 (TM1), F200 (TM5), K204 (TM5), A207 (TM5), L210 (TM5), I211 (TM5), I280 (TM7), I284 (TM7), and L291 (TM7), while L86 (TM2), W89 (TM2), G93 (TM2), V94 (TM2), V98 (TM2), P275 (TM7), I279 (TM7), P283 (TM7), L287 (TM7), V290 (TM7) correspond to binding site II (Figure 6E).

## 6. LAT1 Structure–Function Studies

**Figure 8:**
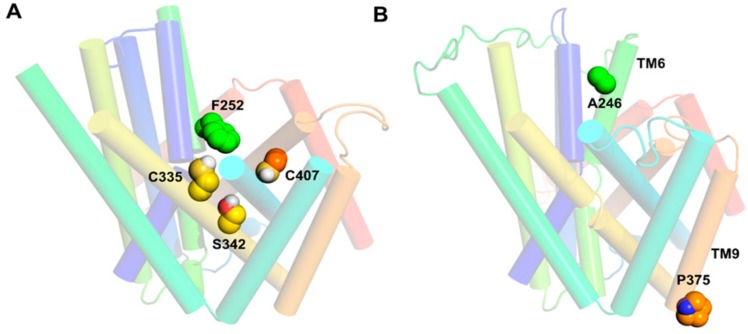
(**A**) Front view of the outward-open model of LAT1 showing residues of the substrate binding site; (**B**) Front view of the outward-occluded model of LAT1 showing residues constituting the missense mutations in patients with autism spectrum disorders (ASD) and motor delay. The residues are shown in space-filling style. The outward-open model of LAT1 was built as described above in MODELLER v9.13 [57] and it is based on the AdiC structure (PDB ID: 3OB6) and the published alignment [52].

Among the different amino acid transporters that have not been crystallized yet, the least is known about structure–function relationships of LAT1. To gain insights into the structure–function relationship, Napolitano et al. [52] selected the active site residues F252, C335, S342, and C407 (Figure 8A) for site-directed mutagenesis. F252 was substituted with a less hydrophobic and conserved residue, corresponding to the AdiC homolog (F252W), and with a non-conservative residue (F252A), altering the length, hydrophobicity, and steric properties of the side chain. Cysteine mutants were generated by substituting C335, C407, or both with the less hydrophobic alanine. The transport activity of the wild type (WT) and mutants were measured in proteoliposomes reconstituted with human LAT1 to evaluate the potential loss of function in the mutants [9,52,76]. F252A substitution completely abolished the transport activity, while F252W showed impaired transport with respect to the WT. The C407A mutant was identical to the WT, whereas S342G, C335A, and the double mutant C335A/C407A showed reduced activity compared to the WT. These results demonstrated that F252 is possibly functioning as a proximal gate element in LAT1 (Figure 6C,D and Figure 8A), which corresponds to the gate residue W202 in AdiC [39], allowing substrate entry to the binding site. The residues S342 and C335 promote substrate binding prior to translocation. Recently, Tărlungeanu et al. [77] identified two LAT1 mutations, A246V and P375L, in patients with autism spectrum disorders (ASD) and motor delay. A246 is located in TM6 in close proximity to the extracellular side and to the substrate translocation pathway (Figure 8B). The mutation of alanine to the bigger valine is predicted to influence the transporter’s structure by disrupting helix–helix packing and ligand transport. In addition, the possible steric hindrance from the side chain of valine may hinder the structural shift of TM6 required for the occlusion of the substrate in the binding site. P375 is located in TM9 in close proximity to the cytoplasmic side (Figure 8B). The mutation of proline to leucine is proposed to disrupt the flexibility required for transport by LAT1. The uptake assays in cells (human dermal fibroblasts) carrying the homozygous A246V or P375L mutations showed significant reduction in l-[^3^H]-leucine uptake compared to the WT, indicating that these mutations reduce LAT1-mediated uptake of branched-chain amino acids into the brain, thus leading to ASD.

## 7. Binding Hypothesis of LAT1 Ligands

**Figure 9:**
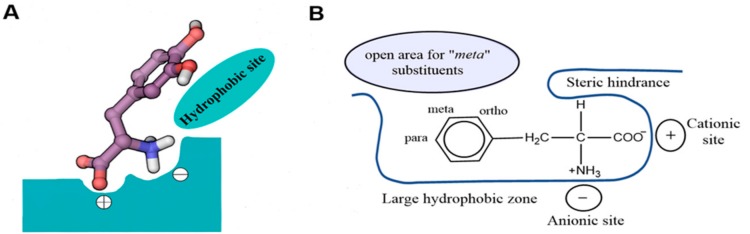
(**A**) The predicted model of the substrate binding site of LAT1 with l-DOPA as a model substrate. The binding site of LAT1 (shown in green color) is proposed to consist of three recognition sites (+, −, and hydrophobic site) that are responsible for the binding of the negatively charged α-carboxyl group, the positively charged α-amino group, and the side chain; (**B**) The binding site model of the Blood–Brain Barrier (BBB) LAT1 in relation to the amino acid phenylalanine. (**A**,**B**) These figures were recreated from references [6,78], respectively.

Uchino et al. [78] first proposed a model for the substrate binding site of LAT1. According to this model, both positive and negative charges at the Cα of the ligand are required for substrate transport. The binding site of LAT1 is proposed to contain specific recognition sites in the peptide backbone that mediate the binding of charged head groups of amino acids through electronic interactions, while the binding of the side chain is presumably mediated by hydrophobic residues (Figure 9A). Smith [6] proposed a similar binding site model for the cerebrovascular LAT1 (Figure 9B) and he concluded that, for LAT1 affinity, substrates must contain (i) an unsubstituted, free Cα-carboxyl group; (ii) an unsubstituted Cα-primary amino group; (iii) either a hydrogen or methyl on the Cα; (iv) a neutral hydrophobic bulky side chain [6].

**Figure 10:**
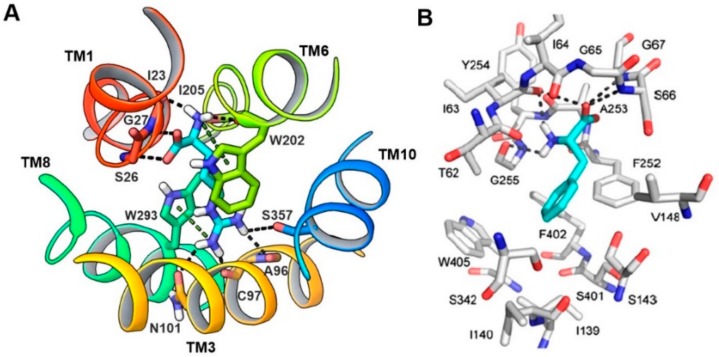
(**A**) Periplasmic view of the substrate binding site of AdiC (PDB ID: 3L1L). Arginine (cyan) is bound to AdiC at the center of the transport path, recognized by amino acids from TM1, TM3, TM6, TM8, and TM10. Arginine and interacting residues of AdiC are shown in stick representation; (**B**) Predicted binding mode of phenylalanine in LAT1. LAT1 (gray) and phenylalanine (cyan) are shown in stick representation. (**B**) Figure adapted after securing permission from reference [44].

**Figure 11:**
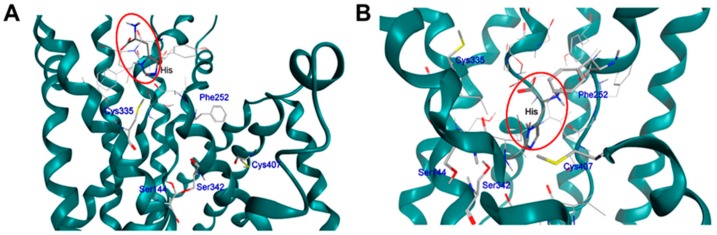
Side view of the predicted binding mode of histidine (enclosed in red oval) in the outward-open (**A**) and outward-occluded (**B**) models of LAT1. LAT1 is shown in ribbon representation; histidine and interacting residues of the binding site are shown in stick representation. This figure was adapted after securing permission from reference [52]. Copyright© 2018 Elsevier B.V.

In 2013, Geier et al. [44] first proposed the binding mode of phenylalanine to LAT1, which revealed that the majority of the polar interactions were conserved between LAT1 and AdiC. In AdiC, the positively charged α-amino group of the arginine donates three hydrogen bonds to the backbone oxygen of I23 in TM1, and W202 and I205 in TM6 (Figure 10A). Additionally, the positively charged α-amino is involved in a cation–π interaction with the indole ring of W202. The α-carboxyl group accepts two hydrogen bonds from S26 and G27 (TM1). At the other end of arginine, the guanidinium group is stacked against W293 in TM8 via a cation–π interaction [39], and the nitrogen atoms of the guanidinium donate hydrogen bonds to A96, C97, N101 in TM3, and S357 in TM10. The corresponding residues interacting with phenylalanine in LAT1 include I63 (TM1), F252 (TM6), and G255 (TM6), accepting hydrogen bonds from the positively charged α-amino group, and S66 and G67 (TM1), donating hydrogen bonds to the negatively charged α-carboxyl group (Figure 10B). Docking studies performed by Napolitano et al. [52] against the LAT1 models showed that residues F252, C335, S342 and C407 were mediating the binding of histidine (Figure 11). Because the α-carboxyl and α-amino groups are conserved among known LAT1 ligands, such as thyroxine (T_4_), 3,3′,5-triiodothyronine (T_3_), gabapentin, melphalan, and l-DOPA, it is highly likely that these compounds may display a similar binding mode in LAT1. From the docking studies performed on LAT1, it can be surmised that residues located near the helix break of TM1 (I63, S66 and G67) and TM6 (F252, G255) are primarily responsible for the substrate binding through backbone hydrogen bond interactions, while residues I139, V148, F252, F402 and W405 likely promote the binding of the side chain via hydrophobic interactions.

**Figure 12:**
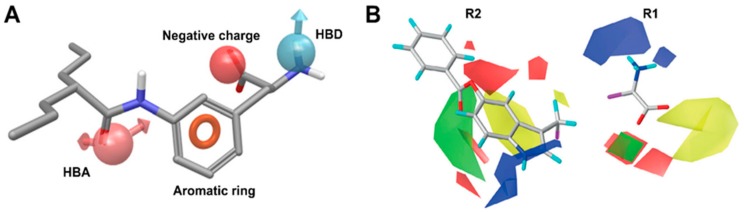
(**A**) 3D pharmacophore model for LAT1 substrates. The model consists of four pharmacophoric features: hydrogen bond acceptor (HBA) (pink, leftmost), aromatic ring (orange), negatively charged group (red), and hydrogen bond donor (HBD) (blue, rightmost); (**B**) Topomer comparative molecular field analysis (CoMFA) model for LAT1-targeting prodrugs. Green and red represent areas where adding steric features and negative charge or hydrogen bond acceptors are favored, respectively. Blue represents areas where a more positive charge or hydrogen bond donors are preferred, whereas yellow contours designate sterically disfavored regions. R1 and R2 indicate the common core, amino acid function and variable topomer, side chain and parent drug. (**A**) This figure was adapted after securing permission from reference [79]. Copyright© 2018 Elsevier B.V. (**B**) This figure was adapted after securing permission from reference [80]. Copyright© 1999–2018 John Wiley & Sons, Inc.

Ylikangas et al. [79] first proposed a pharmacophore-based binding hypothesis for LAT1 substrates. In this study, a ligand-based 3D pharmacophore model was generated from a set of 28 LAT1 substrates. The pharmacophore model is based on four features: hydrogen bond acceptor (HBA), hydrogen bond donor (HBD), negative charge, and aromatic ring (Figure 12A). The structure–activity relationship (SAR) analysis of substrates in the context of the pharmacophore model revealed that negatively charged and HBD features are essential for good affinity, and removal or modifications of either of those result in a weak affinity or complete inability of the ligand to bind to LAT1. Because planar aromatic compounds were found more potent than the equally lipophilic nonplanar structures, the aromatic feature was considered as a superior feature than lipophilicity to increase ligand affinity. The HBA feature, following the aromatic region, was found to improve the binding of LAT1 substrates. In another study, Ylikangas et al. [80] performed a three-dimensional quantitative structure–activity relationship (3D-QSAR) study of 39 LAT1 binding compounds using classical and topomer comparative molecular field analysis (CoMFA) [81,82]. The CoMFA model showed that the contribution of steric interactions was stronger than the effect of electrostatics in two topomers, R1: amino acid terminal and R2: side chain, prodrug bond, and parent drug, respectively (Figure 12B). The model indicated that addition of steric features above the aromatic plane of the amino acid chains and in regions beyond the amino acid terminal (yellow contour in R1, Figure 12B) decreases the affinity. Increasing the positive charge near the amine function (blue contours at R1) and adding a negative charge near the carboxylic acid (red cubic-shaped contours at R1) is beneficial for LAT1 affinity. In addition, the model showed that the moiety for the design of prodrugs should be relatively planar because large, branched substituents can reach areas that can decrease the affinity (yellow contour in R2). The addition of steric moieties near the 5- and 6-positions of l-tryptophan is favorable for ligand binding, as depicted by a green crescent-shaped contour at R2 (Figure 12B). These positions are equivalent to the 3- and 4-positions of l-phenylalanine. Thus, these positions can be utilized to attach the parent drug to the prodrug moiety. Furthermore, an increase in the negative charge over the aromatic ring of l-phenylalanine and l-tryptophan can enhance the affinity of compounds. The SAR-driven ligand-based pharmacophore modeling and 3D-QSAR study have elucidated critical molecular features and their 3D arrangement required to achieve high affinity in LAT1. These ligand-based models can serve as guidelines for the rational design of novel ligands of LAT1.

## 8. LAT1 Ligand Discovery

**Figure 13:**
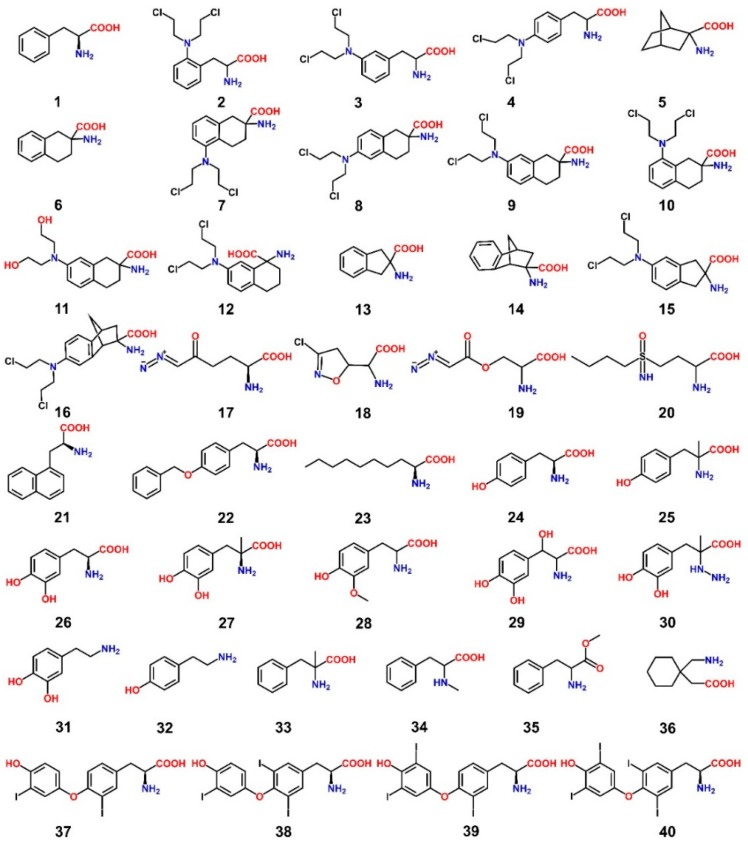
**1**, l-Phenylalanine **2**, *o*-Sarcolysin **3**, *m*-Sarcolysin **4**, Melphalan **5**, 2-Amino-2-norbornanecarboxylic acid (BCH) **6**, (±)-2-Amino-1,2,3,4-tetrahydro-2-naphthoic acid **7**, dl-2-NAM-5 **8**, dl-2-NAM-6 **9**, dl-2-NAM-7 **10**, dl-2-NAM-8 **11**, dl-dechlorinated-NAM **12**, dl-1-NAM-7 **13**, (±)-2-Aminoindane-2 carboxylic acid **14**, (±)-2-Aminobenzo-bicyclo-[2.2.1]heptane-2′-exo-carboxylic acid **15**, (±)-2-amino-(*bis*-2-chloroethyl)-5-aminoindane-2-carboxylic acid **16**, (±)-2-endo-amino-*bis*(2-chloroethyl)-7′-aminobenzobicyclo[2.2.1]heptane-2-exo-carboxylic acid **17**, l-6-diazo-5-oxo-norleucine (l-DON) **18**, Acivicin **19**, Azaserine **20**, Buthionine Sulfoximine (BSO) **21**, l-1-naphthylalanine **22**, *o*-benzyl-l-tyrosine **23**, l-2-amino-nonanoic acid **24**, l-Tyrosine **25**, α-methyltyrosine **26**, l-DOPA **27**, α-methyldopa **28**, 3-*o*-methyldopa **29**, Droxidopa **30**, Carbidopa **31**, Dopamine **32**, Tyramine **33**, α-methylphenylalanine **34**, *N*-methylphenylalanine **35**, Phenylalanine methyl ester **36**, Gabapentin **37**, 3,3′-diiodothyronine **38**, l-T_3_**39**, 3′,5′,3-triiodothyronine (r l-T_3_) **40**, l-T_4_

The early discovery of LAT1-targeting ligands started with the aim to find new chemotherapeutic agents that can penetrate the BBB and work in combination with other anticancer drugs, such as the nitrosoureas, for the treatment of brain tumors [6]. Most anticancer drugs have minimal access to the CNS due to limited BBB passive diffusion, aggressive active BBB efflux, or high plasma protein binding, necessitating novel means of enhanced chemotherapeutic drug delivery to target brain tumors [6]. Of all the influx transporters at the BBB, LAT1 is well suited as a brain drug delivery vector [83] by altering CNS impervious drugs in a way such that they become substrates of LAT1 and show improved BBB penetration [84,85]. Table 2 summarizes the validation methods and biological activity values of the LAT1 ligands that are discussed below. The alkylating phenylalanine mustards **2**–**4** (Figure 13), belonging to a group of antitumor agents, were first synthesized in the 1950s [86,87,88] and were found to possess a broad range of activity against both experimental [89] and human neoplasms [90]. These antitumor agents incorporated into their structure the LAT1 substrate phenylalanine **1** and the cytotoxic *bis*(2-chloroethyl)amino group. Considering the substitution pattern of the phenylalanine, the cytotoxic activity (LD_10_) in in vivo tumor models showed the following trend: *ortho*-substituted **2** > *meta*-substituted **3** > *para* compound **4**. The l-forms of phenylalanine mustards showed increased LD_10_ compared to the d-isomers [91]. In search of a new scaffold for inhibitors of LAT1, Vistica et al. [92] tested a series of cyclic amino acids for transport affinity in tumor cells and discovered compound **6** as a potent competitive inhibitor of LAT1. This scaffold was later utilized to selectively introduce the *bis*(2-chloroethyl)amino group to obtain derivatives **7**–**10**, among which compound **9** was found to be an extremely potent competitive inhibitor of LAT1 in murine L1210 leukemic cells (*K*_i_ = 0.2 μM) [93]. In addition, compound **9** possessed enhanced in vitro antitumor activity in murine L1210 leukemic cells and reduced myelosuppressive activity in bone marrow cells of male CDF_1_ mice when compared to its prototype melphalan **4** [93]. Kinetic studies using the in situ rat brain perfusion technique [94] revealed that the brain uptake of compound **9** was 40-fold greater than that of compound **4** [95]. Moreover, the brain influx of compound **9** was saturable, sodium-independent, and inhibited by compound **5** [95,96], indicating LAT1-mediated transport. Takada et al. [97], by using the in situ rat brain perfusion technique, showed that compound **11** had approximately 10 times less affinity than compound **9**, but both of them had similar low *V*_max_, indicating that compound **9** was not alkylating LAT1. The other positional isomer **7** showed 100-fold less affinity than compound **9**, while compounds **8** and **10** exhibited more than 1000-fold less affinity than compound **9**, indicating that the binding affinity for BBB LAT1 is dependent not only upon side chain hydrophobicity but also upon the 3D position of the side chain relative to the amino acid moiety. In line with this, compound **3** showed a 100-fold greater affinity for LAT1 and was taken up into the rat brain during perfusion at >50-fold rate than compound **4**, confirming the preference for substituents in the *meta* position of the phenylalanine ring. Moreover, the transfer of the amino acid group in compound 9 from C-2 to C-1 in compound **12** diminished LAT1 affinity by over three orders of magnitude (*K*_i_ = 730 μM). Later, Matharu et al. [98] assessed the LAT1 affinities of more rigid analogs, compounds **13**–**16**, through competitive l-[^14^C]-leucine uptake inhibition using the in situ rat brain perfusion technique. The results indicated that a restriction of the conformational flexibility of the scaffold **6** appeared to be detrimental to LAT1 binding, since the indane analog **15** and the bicyclo analog **16** had 60 and 25 times less affinity, respectively, for LAT1 than compound **9**, but an approximately matched affinity to compound **7**. However, both compounds **15** and **16** were superior ligands than compound **4**. The rigid scaffolds **13** and **14** had approximately two and three times less affinity than compound **6** for LAT1. Takada et al. [95] screened five anticancer agents, namely, compounds **4**, **17**–**20**, for BBB LAT1 affinity by evaluating their ability to inhibit the uptake of l-[^14^C]-leucine using the in situ rat brain perfusion technique. The results demonstrated that these compounds do not have strong transport affinity (1/*K*_i_ = 0.2–11 mM^−1^) for BBB LAT1 as compared to compound **1** (~100 mM^−1^). Many diverse compounds were tested for affinity to ascertain the tolerability of side chain size in LAT1. For example, compounds **21**–**23** were found to exhibit high affinity for LAT1 (*K*_m_ or *K*_i_ < 10 μM; apparent affinity > 100 mM^−1^). l-T_3_ **38** also showed high affinity for cerebrovascular LAT1 (*K*_i_ = 1.0 ± 0.1 μM, apparent affinity = 1000 mM^−1^), although rat brain uptake of compound **38** is additionally mediated by the cerebrovascular thyroid hormone transporter. These results indicated that several large amino acid analogs could bind to LAT1. However, the maximal transport capacity decreases with amino acid size [97]. Uchino et al. [78] arrived at a similar conclusion after their study showed that compounds **38**, **40**, and **4** inhibited the l-[^14^C]-phenylalanine uptake in human LAT1−4F2hc expressing *Xenopus laevis* oocytes, implying that the substrate binding site of LAT1 can accommodate large side chains, such as those of thyroid hormones (THs) and melphalan. Nonpolar interactions between the side chain of compound **38** and the binding site was considered as a strong contributing factor for the high affinity of **38** (*K*_i_ = 5.8 μM) in LAT1. Their study showed that compounds **34** and **35** had little effect on LAT1-mediated uptake, suggesting that both α-amino and α-carboxyl groups are recognized by LAT1. In agreement with this, compounds **31** and **32**, both of which lack α-carboxyl groups, and compound **30** failed to inhibit LAT1-mediated transport. Compounds **25**, **27** and **33** behaved like LAT1 substrates, indicating that the binding site of LAT1 can accommodate methyl substituents on the α-carbon. Authors, therefore, suggested that substrate binding is dependent on the interaction between the charged α-amino and α-carboxyl group with the binding site and that an interaction between the α-carbon and the binding site is not essential. This hypothesis was coherent with the observation that compound **36**, which is not a conventional amino acid, is a substrate of LAT1. The failure of compound **29** to interact with LAT1 was attributed to the possible interference of the β-hydroxyl group with the substrate binding site and to an intramolecular hydrogen bonding interaction between the β-hydroxyl group and the α-carboxyl group, resulting in loss of electron density on the carboxyl. The LAT1 affinity of compounds **1**, **24**, and **26** varied with the number of phenolic hydroxyl groups on the aromatic ring, that is, *K*_i_ had the trend: **26** (2 OH) < **24** (1 OH) < **1** (0 OH). The *K*_i_ trend **24** > **28** > **26** was consistent with the observed trend for calculated ClogP values 0.0984 > −0.0524 > −0.4986, indicating that lipophilicity is an important determinant for the binding of the side chains of ligand in LAT1 [99,100]. Based on the Connolly accessible area and ClogP calculation for the compounds studied, authors concluded that a LAT1 substrate becomes an inhibitor when its Connolly accessible area becomes larger (> 500 Å^2^) and/or has a calculated ClogP > 2.0, such as for compounds **4**, **38**, and **40** that behaved more like inhibitors though transported at low rates [78]. Friesema et al. [101] measured the uptake of THs (**37**–**40**) in human LAT1−4F2hc expressing *X. laevis* oocytes. The experiments demonstrated the uptake trend **37** > **38**~**39** > **40**. The K_m_ of compounds **37**–**40** was 7.9 μM, 0.8 μM, 12.5 μM, and 7.9 μM, respectively. In contradiction to LAT1, LAT2 allows the uptake of compounds **37** and **38**, but not of compounds **39** and **40**. Additionally, THs are not at all exported by LAT2 [102]. This might indicate that the binding site of LAT1 is large in size as compared to LAT2, facilitating the transport of bulky ligands such as **40**.

**Figure 14:**
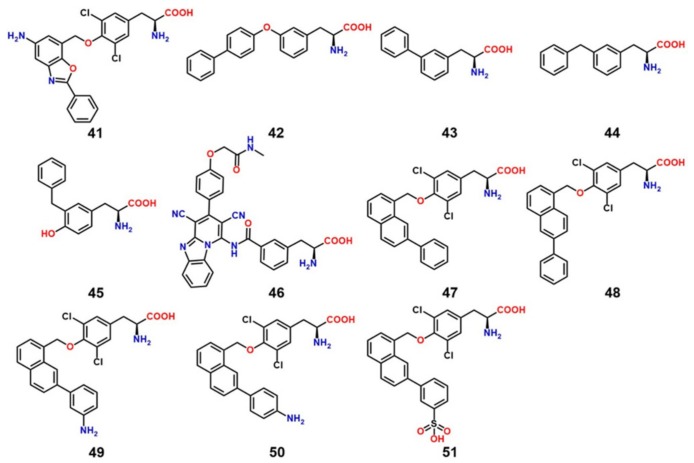
Chemical structures of LAT1 inhibitors (**41**–**47**, **49**–**51**); Compound **48** failed to inhibit LAT1 in the tested concentration range [109]. Compounds **41**, KYT-0353 **42**, KYT-0284 **43**, 3-([1,1′-biphenyl]-3-yl)-2-aminopropanoic acid **44**, 2-amino-3-(3-benzylphenyl)propanoic acid **45**, 2-amino-3-(3-benzyl-4-hydroxyphenyl)propanoic acid **46**, KMH-233 **47**, SKN101 **48**, SKN102 **49**, SKN103 **50**, SKN104 **51**, SKN105.

**Figure 15:**
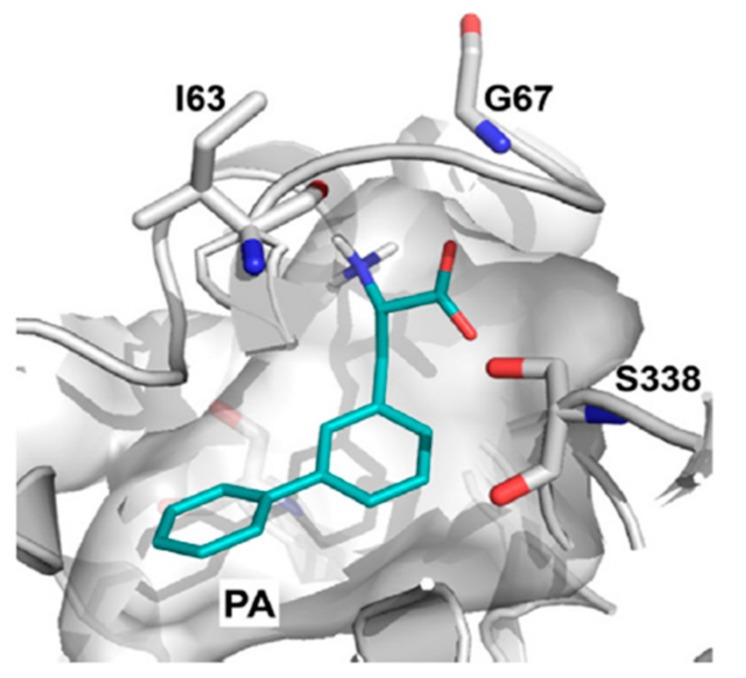
Predicted binding mode of inhibitor **43** in the substrate binding site of LAT1. This figure was adapted after securing permission from reference [107]. Copyright© 2018 Elsevier B.V.

The discovery of LAT1 inhibitors has relied largely on the identification of new compounds that can mimic LAT1 substrates, thus competing for target binding [28]. However, the compounds identified through this approach require high concentration to produce biological effects, e.g., BCH (~10 mM). BCH inhibits both LAT1 and LAT2, thus it is not LAT1-selective [103]. l-T_3_, as discussed before, is a high-affinity LAT1 ligand and has a meager transport rate, making it a LAT1-selective inhibitor [78]. During the last decade, several new low-micromolar LAT1 selective inhibitors have emerged from different studies. Oda et al. [104] discovered compounds **41** and **42** (Figure 14), both of which strongly inhibit LAT1. Compound **41** is a novel tyrosine analog with high LAT1 selectivity and possesses potent in vitro and in vivo inhibitory activity. It showed dramatic inhibition of l-[^14^C]-leucine uptake in both S2 LAT1 (IC_50_ = 0.14 μM) and human colon cancer (HT-29) LAT1 (IC_50_ = 0.06 μM) cells. It also inhibited cell growth (IC_50_ = 4.1 μM) in HT-29 cancer cells, S2 cells (IC_50_ = 16.4 μM) [104], human oral cancer cells (YD-38) [105], and leukemic cells [106]. It inhibited leucine uptake in human LAT2 expressing S2 cells with an IC_50_ > 10 μM and inhibited cell growth in S2 human LAT2 at a very high concentration (IC_50_ > 1000 μM). It is currently undergoing a Phase I clinical trial in humans (UMIN000016546) as a novel adjuvant treatment approach for solid tumors. Compound **42** inhibited leucine uptake in both LAT1- (IC_50_ = 2.0 μM) and LAT2- (IC_50_ = 0.45 μM) expressing S2 cells, thus it is not LAT1-selective. Augustyn et al. [107] identified compounds **43**–**45** that inhibited [3H]-gabapentin uptake in HEK human LAT1 cells, with an IC_50_ of 6.6, 7.3, and 9.1 μM, respectively. Among all compounds, **43** with a phenyl at *meta* position, strongly inhibited LAT1. The *ortho* and *para* analogs of phenylalanine were found to be inferior substrates relative to the *meta* isomer. Through SAR-guided computational modeling, it was proposed that large lipophilic groups, such as phenyl in **43**, cause an increase in LAT1 affinity due to hydrophobic interactions with a subpocket PA (Figure 15) at the expense of a decreased transport capacity (lower *V*_max_), resulting in compounds that behave more like inhibitors rather than substrates. The prediction of a hydrophobic subpocket in LAT1 might explain the high affinity of compounds **42**, **44** and **45**, where bi-phenyl, in **42**, and benzyl, in **44** and **45**, seem to satisfy the PA. Similarly, **41** has a large lipophilic group at the *para* position which can rotate to mimic a *meta* conformation and fill the PA. Huttunen et al. [108] identified a selective and non-transportable LAT1 inhibitor **46** based on a 3D-QSAR model of the rat LAT1 binding site [85]. Compound **46** inhibited leucine uptake in MCF-7 cells with an IC_50_ of 18.2 μM but failed to inhibit l-alanine uptake in LAT2 within the concentration range of 50–1000 μM, indicating exclusive selectivity for LAT1. The compound also showed significant reduction of cell growth with an IC_50_ value of 124 ± 24 μM. Additionally, the inhibitor was found to reduce the growth of MCF-7 cells effectively alone but also in combination with the aminopeptidase inhibitor bestatin and the DNA cross-linking agent cisplatin, at low concentrations. In a recent study, Kongpracha et al. [109] designed a novel series of LAT1 inhibitors, **47**–**51**, based on the structure of T_3_. Compounds **47**, **49**–**51**, having substitution at the C-7 position of the naphthalene ring, selectively inhibited LAT1-mediated l-[14C]-leucine uptake in HEK293 cells but had no effect on the LAT2-mediated l-[14C]-alanine uptake in HEK293 cells. Compound **48**, with a phenyl group at C-6 position, failed to inhibit LAT1 in the tested concentration range, suggesting that modifications at the C-6 position are not favorable for the interaction with LAT1. Compound 49 was considered to be a non-transportable blocker rather than a substrate of LAT1 and inhibited LAT1 in a competitive manner with an IC_50_ of 1.98 ± 1.07 μM and a *K*_i_ of 2.1 ± 0.12 μM. Compound **49** also suppressed mTOR activity and the growth of PANC-1 cells. Moreover, compound **49** in combination with cisplatin additively enhanced growth inhibition in PANC-1 and H520 cells. The SAR information provided by these compounds might be useful in ligand-based and rational drug design of potent LAT1 inhibitors.

## 9. LAT1-Mediated Prodrug Delivery

**Figure 16:**
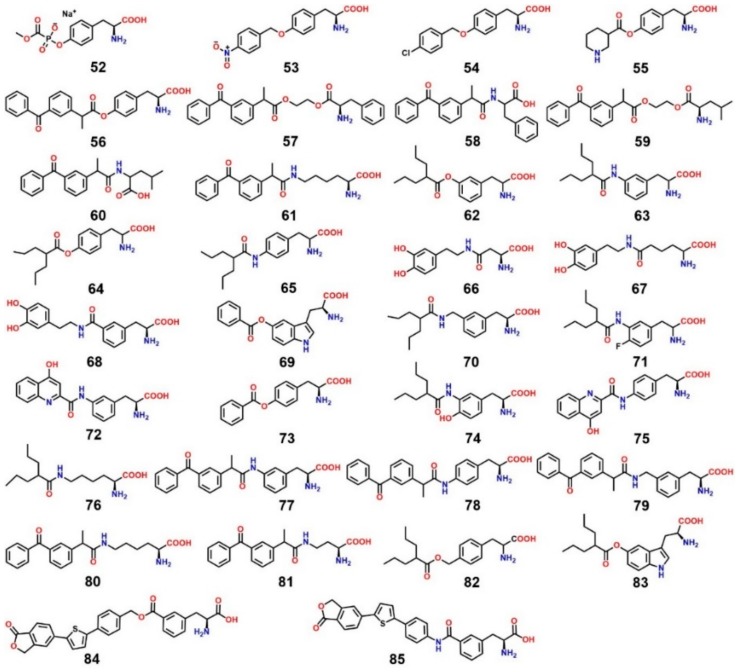
Chemical structures of LAT1-targeting prodrugs; Compounds **57**–**60** failed to inhibit LAT1 [114].

Several prescription drugs, such as l-DOPA [110], baclofen [111], gabapentin [112], and melphalan [113], have been shown to be transported into the brain via cerebrovascular LAT1, demonstrating the utility of LAT1 in CNS drug delivery. These drugs have strong structural similarity to endogenous substrates of LAT1. LAT1-targeting prodrugs share the same structural resemblance and consist of a parent drug attached to the side chain of the amino acid through a biodegradable linkage, and an unsubstituted α-carboxyl and α-amino group to achieve effective LAT1 binding [78,79,114]. Several studies have successfully exploited LAT1 for the design and development of prodrugs. For example, Walker et al. [115] identified a phosphonoformate l-tyrosine conjugate **52** (Figure 16) which inhibited the transport of l-[^3^H]-tyrosine in porcine brain microvessel endothelial cells. In another study, *p*-nitro- and *p*-chlorobenzyl ether conjugates of l-tyrosine **53** and **54** inhibited the transport of l-[^3^H]-tyrosine in a rabbit corneal cell line [116]. An in vivo experiment with a genetically seizure-prone strain (DBA/2) of mice demonstrated that the tyrosine derivative of nipecotic acid **55** is transported into the brain via LAT1 [117]. Gynther et al. [114], by using the in situ rat brain perfusion method, showed that ketoprofen conjugated to the phenolic hydroxyl group of l-tyrosine **56** is transported into the brain and shows high affinity for LAT1. Ketoprofen prodrugs conjugated with the carboxyl group **57**, **59** or the amino group **58**, **60** of the phenylalanine or leucine failed to bind to LAT1, suggesting that a putative substrate should have an α-amino and an α-carboxyl group and a nonpolar side chain [114]. In a study, LAT1-mediated brain uptake of ketoprofen–l-lysine conjugate 61 was demonstrated through the in situ rat brain perfusion technique [91]. Peura et al. [118] reported that valproic acid derivatives **62** and **63**, substituted at *meta* position of phenylalanine, bind to LAT1 with a higher affinity (~10 times) compared to the *para*-substituted derivatives **64** and **65**. The in situ rat brain perfusion experiments revealed that the rat brain uptake of the *meta* prodrug was 2-fold higher compared with those of the *para*-substituted derivatives [118]. In another study, Peura et al. [119], by using the in situ rat brain perfusion technique, showed that dopamine attached to the *meta *position of phenylalanine **68** exhibited sufficient affinity for LAT1 for carrier-mediated brain uptake. The aspartic acid and adipic acid prodrugs **66** and **67** were not found to possess significant affinity for LAT1. Ylikangas et al., evaluated seven prodrugs, **69**–**76**, based on the CoMFA model described earlier using the in situ rat brain perfusion technique. The benzoic acid prodrug **69** substituted at the 5-position of l-tryptophan showed 99% l-leucine uptake inhibition [80]. Recently, Puris et al. [120] designed three prodrugs of ketoprofen conjugated in *meta*- or *para*-position of phenylalanine, **77**–**79**, and two ketoprofen prodrugs, **80** and **81**, using aliphatic promoieties. The in situ rat brain perfusion technique demonstrated that prodrugs **77**–**79** significantly inhibited the cellular uptake of l-[^14^C]-leucine (91.4 ± 1.6%, 75.7 ± 1.8% and 88.3 ± 2.3%, respectively), while prodrugs **80** and **81** showed minor reduction of l-[^14^C]-leucine uptake (17.6 ± 6.7% and 6.2 ± 2.2%, respectively). In a study using the in situ rat brain perfusion technique, valproic acid prodrugs **82** and **83** and perforin inhibitor prodrugs **84** and **85** showed 16%, 81%, 97.4% and 59.4% inhibition of l-[^14^C]-leucine uptake, respectively [121,122]. The studies described here have demonstrated that natural substrates of LAT1 can serve as an excellent template to enhance the delivery of small molecules into the brain.

## 10. Structure-Based Ligand Discovery of LAT1

**Figure 17:**
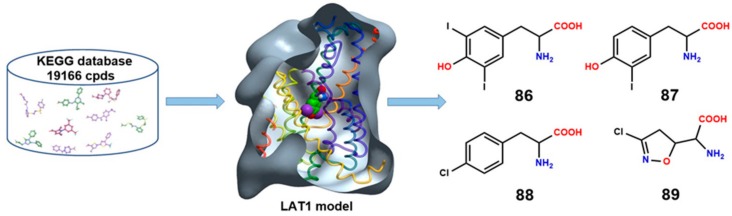
Virtual screening for novel LAT1 ligands through structure-based methods that involved docking of database compounds into the binding site of a LAT1 homology model. Four new ligands were discovered: **86**, 3,5-diiodo-l-tyrosine; also shown in space-filling representation bound to the outward-occluded model of LAT1 **87**, 3-iodo-l-tyrosine **88**, fenclonine, and **89**, acivicin.

As far as we know, there is only a single study by Geier et al. [44] that describes the discovery of novel ligands through docking studies using LAT1 models. In this study, docking was first performed to evaluate the ability of the different LAT1 models built on AdiC and ApcT to discriminate between known LAT1 ligands and decoy molecules (i.e., compounds with physical properties similar to those of known ligands, but with different topology). Decoys were generated using the Directory of Useful Decoys Enhanced (DUD-E) [123]. The enrichment calculations provide an estimate whether a model could be used for prospective virtual screening, as well as if it might serve for structure-based drug design. The area under the curve (logAUC) score of the final refined model of LAT1 was 31.9, indicating that it could be used for the prediction of ligands for experimental testing. Finally, virtual screening of drug and metabolite libraries from Kyoto Encyclopedia of Genes and Genomes (KEGG) [124] was performed against this model through docking, using DOCK 3.5.54 [125]. Docking poses of the highest-ranked compounds were inspected visually to prioritize compounds for experimental testing. Twelve of the top-scoring molecules were experimentally tested by *cis*-inhibition, among which four compounds, **86**–**89** (Figure 17), inhibited [3H]gabapentin uptake in HEK-LAT1 cells. The IC_50_ of compounds **86** and **89** was 7.9 and 340 μM, respectively. Compound **86** showed 25% higher inhibition than **87**. *Tran*s-stimulation experiments showed that **86** and **87** were inhibitors, whereas **88** and **89** were LAT1 substrates. The two compounds, **87** and **89**, also revealed to decrease proliferation of a GBM cell line. In this elegant study, four novel LAT1 ligands were identified, emphasizing the reliability of AdiC-based LAT1 models in structure-based ligand discovery.

## 11. Covalent Inhibition of LAT1

The major drawback of competitive noncovalent inhibitors is their reduced potency caused by the high concentration of endogenous substrates that compete for target binding [126]. Covalent inhibitors, in comparison to classical inhibitors, display nonequilibrium binding kinetics that limits the competition with high endogenous substrate concentrations for target binding, e.g., endogenous ATP in relation to covalent kinase inhibitors [127]. The major advantage of covalent inhibitors is that they provide potent inhibition of the target at low doses, with prolonged duration of action. Because the endogenous substrates have high affinity (*K*_m_ = 15–50 μM) for LAT1, it is desirable to identify covalent ligands that show minimum competition with amino acids for the binding site. A covalent ligand usually consists of an electrophilic moiety that forms a covalent bond with the thiolate group of cysteine in the binding site, resulting in a non-specific reaction leading to the inhibition of the transport activity. Because of the high expression of LAT1 in cancers and of its critical role in cancer growth, covalent inhibition could provide an efficient way to suppress malignant tumors.

**Figure 18:**
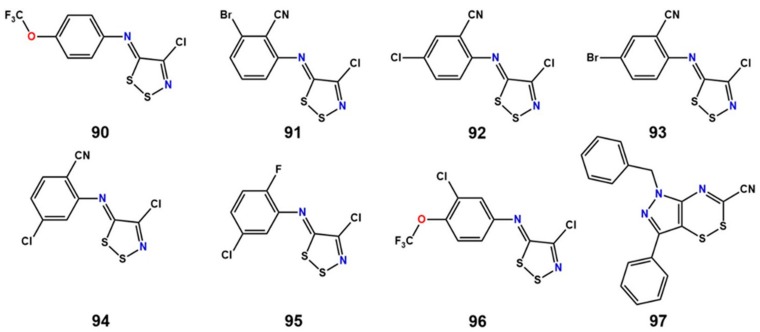
Chemical structures of the most potent irreversible covalent inhibitors of LAT1.

**Figure 19:**
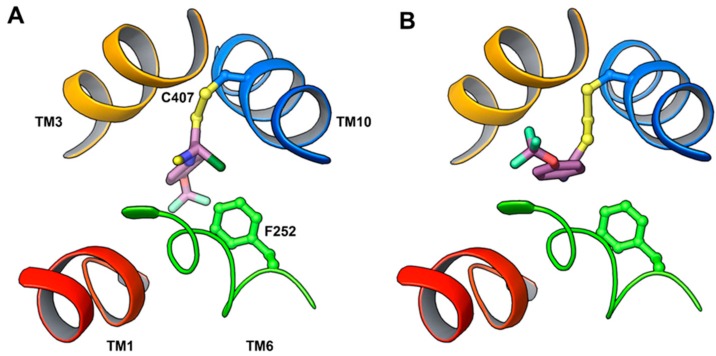
Predicted binding mode of compound **90** (violet) in the outward-open model of LAT1 showing disulfide (**A**) and trisulfide (**B**) bond formation between the ligand and C407. The gate residue F252 is in the vicinity of the covalently bound ligand. Reactive docking of compound **90** against the outward-open model of LAT1 was performed using CovDock [132].

Studies have provided evidence that LAT1 can be inactivated by mercurial compounds through covalent inhibition. Boado et al. [128] demonstrated that rabbit LAT1 is highly sensitive to inhibition by inorganic mercury. Site-directed mutagenesis showed that C439 plays a unique role in either LAT1 folding or LAT1 insertion in the plasma membrane. It may also be the key cysteine of LAT1 that is blocked by inorganic mercury, or, alternatively, inorganic mercury may inhibit LAT1 transporter activity via the combined inhibition of multiple cysteine residues. The C439 of rabbit LAT1 corresponds to C443 (TM11) of human LAT1. Napolitano et al. [52] also observed strong inhibition of LAT1 by sulfhydryl (SH) reagents such as methyl-Hg, ethyl-Hg, aminoethyl methanethiosulfonate (MTSEA), and *N*-ethylmaleimide (NEM). Mutational studies excluded the involvement of C335 and C407 in the inhibition that are located in close proximity to the substrate binding site. Kinetic studies showed that inhibition of LAT1 by HgCl_2_ occurs in a non-competitive pattern, indicating an interaction of mercury with residues far from the substrate binding site. The inhibition via mercury was found to be reversed by the S–S reducing agent dithioerythritol (DTE), suggesting the involvement of cysteine(s) other than C335 or C407 in the inhibition. Very recently, Napolitano et al. [129] provided a breakthrough in identifying 1,2,3-dithiazole and 1,2,4-dithiazine-based covalent inhibitors of LAT1 (Figure 18, **90**–**97**). These compounds showed inhibition higher than 90% at 100 μM concentration of human LAT1 reconstituted in proteoliposomes. The IC_50_ value of compounds **90**–**97** was 0.98 ± 0.10, 1.33 ± 0.33, 1.62 ± 0.43, 2.10 ± 0.58, 1.62 ± 0.30, 0.89 ± 0.33, 1.87 ± 0.09, and 5.2 ± 0.11 μM, respectively. The most potent compounds, **90** and **95**, also caused cell death in high-LAT1-expressing SiHA cells, indicating a cytotoxic activity of these inhibitors. The inhibition caused by these compounds continued after removal of the compounds from the reaction mixture, suggesting an irreversible mode of action of these agents. The inhibition was reversed by addition of DTE, indicating the formation of disulfide bonds between the compound and cysteine residues. The site-directed mutagenesis study showed the involvement of C407 in the covalent interaction with the inhibitors. The 1,2,3-dithiazole analogs are predicted to inhibit LAT1 by the formation of an intermolecular disulfide (Figure 19A) or trisulfide bond (Figure 19B) with the thiolate group of cysteine(s). Some of these compounds were also reported as strong inhibitors of the rat isoform of ASCT2 [130,131]. The dithiazole- and dithiazine-based ligands can be useful for designing potential covalent drugs against cancer.

## 12. Conclusions and Future Outlooks

Our knowledge about the structure and function of LAT1 has progressed rapidly in the last few decades, as the biological importance of this transporter has become apparent. Among the system L transporters, LAT1 is the most extensively overexpressed in cancers, making it a valuable drug target. The abundant localization and high expression of LAT1 at the BBB has made it a target of interest for cerebrovascular drug delivery because of its capability to specifically uptake various prescription drugs. Experimental and ligand-based studies have demonstrated that unsubstituted α-carboxyl and α-amino groups and a neutral hydrophobic side chain are indispensable features for LAT1 recognition. A compound substituted at the α-amino or α-carboxyl moiety cannot bind to LAT1. However, modification of the size of the side chain seems allowed and possibly represents a determining factor to achieve affinity through hydrophobic interactions. Recently, a study has shown that charges on the polar head group of ligands are not necessary for binding, and that it is possible to substitute the ligand’s α-carboxyl with hydroxamic acid, although with a reduced effect on efflux rate and gabapentin inhibition [133]. The prediction of a hydrophobic vestibule in LAT1 offers a structure-based explanation for the high affinity of *meta*-substituted phenylalanine analogs. The lipophilic moiety on *meta* position seems to occupy this pocket, thus leading to enhanced hydrophobic interactions and consequently to high affinity. The high affinity of the *para*-substituted analogs KYT-0353, SKN101, and SKN103-105 might be explained by the presence of a bulky lipophilic core at the *para* position, which can twist around the two flexible rotatable atoms (e.g., O and CH_2_ groups) to assume a conformation approximating that of a *meta*-group, finally occupying the hydrophobic pocket.

Ligands such as l-T_4_, l-T_3_, reverse l-T_3_, 3,3′-diiodothyronine 3,5-diiodo-l-tyrosine, and 3-iodo-l-tyrosine might be involved in halogen bond (X-bond) interaction of polarized halogens, contributing substantially to the binding affinity for LAT1. X-bond interaction is an electrostatically driven molecular interaction, where halogens (iodine, bromine, chlorine) exhibit an electropositive crown, or σ-hole, that serves as a Lewis acid to attract a variety of electron-rich Lewis bases (oxygen, nitrogen, and sulfur), in an analogous fashion to classical hydrogen bond interactions [134]. Halogen substituents are amphipathic, capable of serving as both X-bond donors to carboxyl/carbonyl oxygen and aromatic pi-systems in the direction of the σ-hole, and hydrogen bond or X-bond acceptor in the perpendicular direction [135,136]. This explains why the binding site of LAT1 consists not only of aromatic residues (F252, Y254, F402, W405), providing the π electrons, but also of polar residues (S143, N258, T345) contributing hydrogens for hydrogen bond interaction. In addition, the carboxyl of E136 and the backbone oxygen of residues in LAT1 may serve as X-bond acceptor. Augustyn and co-workers have demonstrated that halogens substituted at *meta* position of phenylalanine show higher inhibition of LAT1 with increasing size of the halogen, i.e., I > Br > Cl > F [107]. This suggests that halogen-substituted analogs might be deriving higher affinity from X-bonding, increase in lipophilicity with halogen size, or both.

The 1,2,3-dithiazole analogs provide potent and irreversible inhibition of LAT1 [129] and ASCT2 [131]. Because LAT1 and ASCT2 function cooperatively to regulate leucine transport and activate the mTOR pathway, these covalent inhibitors can be potentially efficacious drugs in suppressing tumor growth by exerting a dual inhibition. Acivicin is a good example that shows dual inhibition of LAT1 and ASCT2 and also binds to metabolic enzymes (e.g., the aldehyde dehydrogenase enzyme family) to inhibit the proliferation of cancer [137,138].

Finally, molecular modeling has again proved to be an invaluable technique for modeling proteins that lack atomic structures, such as LAT1. The structure–function study utilizing an AdiC-based LAT1 model has enriched our understanding of the molecular transport mechanism by characterizing critical residues that are responsible for substrate binding and translocation. In particular, F252 has been revealed as a proximal gate residue of LAT1, responsible for occluding substrates from the periplasm. Through the use of LAT1 models and structure-based methods, novel ligands have been discovered, and insight into the conformational states of the transporter (outward-open, outward-occluded, and inward-open) and into aspects of the protein–ligand interaction and of the potential binding mode has been gained. SAR-guided experimental studies have led to the identification of many LAT1 selective inhibitors, among which KYT-0353 has emerged as a promising and robust compound undergoing clinical trials. Hit-to-lead optimization of the discovered chemotypes may lead to even superior ligands.

LAT1 has been extensively investigated for decades, but challenges remain for targeting this transporter. Despite the crystallization of ED of 4F2hc, little is known about the intermolecular interactions between LAT1 and 4F2hc, and how these two units of the heterodimer regulate the transport cycle. Considering the pharmacological importance of LAT1, the atomic structure of this transporter is urgently needed. The X-ray structure of LAT1 with prototype ligands such as leucine or phenylalanine will reveal the architecture of the transporter, facilitate the understanding of the transport mechanism, and unravel the binding mode of ligands. Additionally, it will provide a robust 3D framework compared to the homology models, for the rational design of inhibitors through structure-based methods and increase the validity of the binding mode hypotheses retrieved from the docking studies on this structure. Also, molecular dynamics (MD) simulations of the putative binding mode of ligands in LAT1 may provide insights into the dynamic nature of the protein–ligand interaction and conformational transitions involved in the substrate translocation cycle.

In conclusion, LAT1 is acknowledged as a cancer-related amino acid transporter, and, in order to target this transporter efficiently, a better understanding of its structural features is required. The studies described in this article have shed light on the molecular features that seem crucial for LAT1 inhibition, and this knowledge could be applied in the rational design of inhibitors. The 3- and 4-position of phenylalanine and 5- and 6-position of tryptophan are critical for LAT1 affinity. Thus, these positions will continue to be exploited for the identification of novel inhibitors and prodrugs. In addition, a structure-based design of non-natural amino acids may help to identify potent ligands of LAT1. It may be expected that computational methods, such as docking, pharmacophore modeling, MD, QSAR, virtual screening, and SAR-driven experimental studies, will play a significant role in the future by providing vital insights into the structural and chemical requirements for the design of LAT1 inhibitors and their development as new anticancer drugs.

## Figures and Tables

**Table 1:** Description of the LAT1 homology models that were built and studied.

Ref./Year	Template	Organism	PDB ID	Resolution (Å)	Conformation	Protomeric Composition	Substrate
[44]/2013	AdiC	*E. coli*	3L1L	3.0	Outward-occluded	Monomer	Arginine
[44]/2013	ApcT	*M. jannaschii*	3GI9	2.48	Inward-open	Monomer	-
[51]/2013	AdiC	*E. coli*	3L1L	3.0	Outward-occluded	Monomer	Arginine
[51]/2013	ApcT	*E. coli*	3GIA	2.32	Inward-open	Monomer	-
[52]/2017	AdiC	*E. coli*	3L1L	3.0	Outward-occluded	Monomer	Arginine
[52]/2017	AdiC	*E. coli*	3OB6	3.0	Outward-open	Homodimer	Arginine
[52]/2017	AdiC	*E. coli*	3LRB	3.61	Outward-open	Homodimer	-

**Table 2:** The validation methods and biological activity values of the LAT1 ligands; a: 1/*K*_i_, b: *K*_i_, c: *K*_m_, d: IC_50_, N/A: data not available.

Cpd	Ref.	Experimental Model	Assay Type	Biological Activity
**1**	[95]	in situ rat brain perfusion	uptake inhibition of l-[^14^C]-leucine	^a^ 95 ± 8 mM
**2**	[91]	in vivo tumor models	cytotoxicity assay	N/A
**3**	[91]	in vivo tumor models	cytotoxicity assay	N/A
**4**	[91]	in vivo tumor models	cytotoxicity assay	N/A
**4**	[93]	Murine L1210 leukemic cells	uptake inhibition of [^14^C]-BCH	^b^ 111.6 ± 7.7 μM
**4**	[98]	in situ rat brain perfusion	uptake inhibition of l-[^14^C]-leucine	^b^ 55 ± 4 μM
**4**	[78]	Human LAT1−4F2hc expressing *Xenopus laevis* oocytes	uptake inhibition of l-[^14^C]-phenylalanine	^b^ 49.1 μM
**5**	[96]	in situ rat brain perfusion	uptake inhibition of l-[^14^C]-leucine	≤^b^ 100 μM
**6**	[98]	in situ rat brain perfusion	uptake inhibition of l-[^14^C]-leucine	^b^ 7.7 ± 0.8 μM
**7**	[98]	in situ rat brain perfusion	uptake inhibition of l-[^14^C]-leucine	^b^ 8.5 ± 0.6 μM
**8**	[98]	in situ rat brain perfusion	uptake inhibition of l-[^14^C]-leucine	^b^ 68 ± 9 μM
**9**	[93]	Murine L1210 leukemic cells	uptake inhibition of [^14^C]-BCH	^b^ 0.22 ± 0.02 μM
**9**	[98]	in situ rat brain perfusion	uptake inhibition of l-[^14^C]-leucine	^b^ 0.079 ± 0.006 μM
**10**	[98]	in situ rat brain perfusion	uptake inhibition of l-[^14^C]-leucine	^b^ 252 ± 44 μM
**11**	[97]	in situ rat brain perfusion	uptake inhibition of l-[^14^C]-leucine	^b^ 1.3 ± 0.01 μM
**12**	[97]	in situ rat brain perfusion	uptake inhibition of l-[^14^C]-leucine	^b^ 730 ± 57 μM
**13**	[98]	in situ rat brain perfusion	uptake inhibition of l-[^14^C]-leucine	^b^ 12.5 ± 1.1 μM
**14**	[98]	in situ rat brain perfusion	uptake inhibition of l-[^14^C]-leucine	^b^ 26 ± 1 μM
**15**	[98]	in situ rat brain perfusion	uptake inhibition of l-[^14^C]-leucine	^b^ 5.0 ± 0.6 μM
**16**	[98]	in situ rat brain perfusion	uptake inhibition of l-[^14^C]-leucine	^b^ 2.1 ± 0.2 μM
**17**	[95]	in situ rat brain perfusion	uptake inhibition of l-[^14^C]-leucine	^a^ 2.7 ± 0.1 mM
**18**	[95]	in situ rat brain perfusion	uptake inhibition of l-[^14^C]-leucine	^a^ 3.4 ± 0.2 mM
**19**	[95]	in situ rat brain perfusion	uptake inhibition of l-[^14^C]-leucine	^a^ 6.2 ± 0.3 mM
**20**	[95]	in situ rat brain perfusion	uptake inhibition of l-[^14^C]-leucine	^a^ 0.21 ± 0.01 mM
**21**	[95]	in situ rat brain perfusion	uptake inhibition of l-[^14^C]-leucine	<^b^ 10 μM
**22**	[95]	in situ rat brain perfusion	uptake inhibition of l-[^14^C]-leucine	<^b^ 10 μM
**23**	[95]	in situ rat brain perfusion	uptake inhibition of l-[^14^C]-leucine	<^b^ 10 μM
**24**	[78]	Human LAT1−4F2hc expressing *Xenopus laevis* oocytes	uptake inhibition of l-[^14^C]-phenylalanine	^b^ 31.1 μM
**25**	[78]	Human LAT1−4F2hc expressing *Xenopus laevis* oocytes	uptake inhibition of l-[^14^C]-phenylalanine	^b^ 107 μM
**26**	[78]	Human LAT1−4F2hc expressing *Xenopus laevis* oocytes	uptake inhibition of l-[^14^C]-phenylalanine	^b^ 67.2 μM
**27**	[78]	Human LAT1−4F2hc expressing *Xenopus laevis* oocytes	uptake inhibition of l-[^14^C]-phenylalanine	^b^ 405 μM
**28**	[78]	Human LAT1−4F2hc expressing *Xenopus laevis* oocytes	uptake inhibition of l-[^14^C]-phenylalanine	^b^ 56.4 μM
**29**	[78]	Human LAT1−4F2hc expressing *Xenopus laevis* oocytes	uptake inhibition of l-[^14^C]-phenylalanine	inactive
**30**	[78]	Human LAT1−4F2hc expressing *Xenopus laevis* oocytes	uptake inhibition of l-[^14^C]-phenylalanine	inactive
**31**	[78]	Human LAT1−4F2hc expressing *Xenopus laevis* oocytes	uptake inhibition of l-[^14^C]-phenylalanine	inactive
**32**	[78]	Human LAT1−4F2hc expressing *Xenopus laevis* oocytes	uptake inhibition of l-[^14^C]-phenylalanine	inactive
**33**	[78]	Human LAT1−4F2hc expressing *Xenopus laevis* oocytes	uptake inhibition of l-[^14^C]-phenylalanine	N/A
**34**	[78]	Human LAT1−4F2hc expressing *Xenopus laevis* oocytes	uptake inhibition of l-[^14^C]-phenylalanine	inactive
**35**	[78]	Human LAT1−4F2hc expressing *Xenopus laevis* oocytes	uptake inhibition of l-[^14^C]-phenylalanine	inactive
**36**	[78]	Human LAT1−4F2hc expressing *Xenopus laevis* oocytes	uptake inhibition of l-[^14^C]-phenylalanine	^b^ 340 μM
**37**	[101]	Human LAT1−4F2hc expressing *Xenopus laevis* oocytes	uptake assay	^c^ 7.9 μM
**38**	[97]	*in situ* rat brain perfusion	uptake inhibition of l-[^14^C]-leucine	^b^ 1.0 ± 0.1 μM
**38**	[78]	Human LAT1−4F2hc expressing *Xenopus laevis* oocytes	uptake inhibition of l-[^14^C]-phenylalanine	^b^ 5.8 μM
**38**	[101]	Human LAT1−4F2hc expressing *Xenopus laevis* oocytes	uptake assay	^c^ 0.8 μM
**39**	[101]	Human LAT1−4F2hc expressing *Xenopus laevis* oocytes	uptake assay	^c^ 12.5 μM
**40**	[101]	Human LAT1−4F2hc expressing *Xenopus laevis* oocytes	uptake assay	^c^ 7.9 μM
**41**	[104]	Human LAT1 expressing S2 cells	uptake inhibition of l-[^14^C]-leucine	^d^ 0.14 μM
**41**	[104]	HT-29 cells	uptake inhibition of l-[^14^C]-leucine	^d^ 0.06 μM
**42**	[104]	Human LAT1 expressing S2 cells	uptake inhibition of l-[^14^C]-leucine	^d^ 2.0 μM
**43**	[107]	Human LAT1 expressing HEK cells	uptake inhibition of [^14^C]-gabapentin	^d^ 7.3 μM
**44**	[107]	Human LAT1 expressing HEK cells	uptake inhibition of [^14^C]-gabapentin	^d^ 6.6 μM
**45**	[107]	Human LAT1 expressing HEK cells	uptake inhibition of [^14^C]-gabapentin	^d^ 9.1 μM
**46**	[108]	Human breast cancer cells (MCF-7)	uptake inhibition of l-[^14^C]-leucine	^d^ 18.2 μM
**47**	[109]	HEK293-Human LAT1 cells	uptake inhibition of l-[^14^C]-leucine	^d^ 1.98 ± 1.07 μM ^b^ 2.1 ± 0.12 μM
**48**	[109]	HEK293-Human LAT1 cells	uptake inhibition of l-[^14^C]-leucine	inactive
**49**	[109]	HEK293-Human LAT1 cells	uptake inhibition of l-[^14^C]-leucine	N/A
**50**	[109]	HEK293-Human LAT1 cells	uptake inhibition of l-[^14^C]-leucine	N/A
**51**	[109]	HEK293-Human LAT1 cells	uptake inhibition of l-[^14^C]-leucine	N/A
